# Design, implementation and evaluation of informal home care support intervention program for lonely older adults in the community: Protocol for a feasibility study

**DOI:** 10.1371/journal.pone.0273924

**Published:** 2022-08-31

**Authors:** Elham Lotfalinezhad, Haidar Nadrian, Ahmad Kousha, Karen Andersen-Ranberg, Mohamed Asghari Jafarabadi, Ahmad Sohrabi, Mina Hashemiparast, Mohammad Reza Honarvar, Shannon Freeman

**Affiliations:** 1 Aging Research Institute, Tabriz University of Medical Sciences, Tabriz, Iran; 2 Department of Health Education and Promotion, Tabriz University of Medical Sciences, Tabriz, Iran; 3 Medical Education Research Center, Tabriz University of Medical Sciences, Tabriz, Iran; 4 Department of Clinical Research, Consultant Physician, Dept. of Geriatrics, Odense University Hospital, University of Southern Denmark, Odense, Denmark; 5 Cabrini Research, Cabrini Health, Malvern, Victoria, Australia; 6 School of Public Health and Preventative Medicine, Faculty of Medicine, Nursing and Health Sciences, Monash University, Clayton, Victoria, Australia; 7 Road Traffic Injury Research Center, Tabriz University of Medical Sciences, Tabriz, Iran; 8 Cancer Control Research Center, Cancer Control Foundation, Iran University of Medical Sciences, Tehran, Iran; 9 Department of Health Education & Promotion, School of Public Health, Zanjan University of Medical Sciences, Zanjan, Iran; 10 Health Management and Social Development Research Center, Golestan University of Medical Sciences, Gorgan, Iran; 11 Faculty of Nursing, University of Northern British Columbia, Prince George, Canada; Prince Sattam Bin Abdulaziz University, College of Applied Medical Sciences, SAUDI ARABIA

## Abstract

**Background:**

Providing lonely older adults with informal home care services is important to improving their health and quality of life. The study aims to evaluate the feasibility of design, implementation and evaluation of an informal home care support intervention program (HoSIP) for community-dwelling lonely older adults in Gorgan, Iran.

**Method/design:**

This feasibility study is a mixed-method with a concurrent nested design. Lonely older adults will be enrolled as the HoSIP intervention group and will receive 12-weeks of informal home care service by peer supporters. The purpose of this feasibility study is to determine the recruitment capability and resulting sample characteristics, data collection procedure and outcome measures, the acceptability and suitability of the intervention and study procedures, the resource and ability to manage the study and intervention, and preliminary evaluation of participant response to intervention. Primary outcomes including participant feelings of loneliness, quality of life, general health, social network, social support, and self-care ability, will be assessed at baseline and post-intervention for the intervention and control groups. Semi-structured interviews will be conducted immediately after the intervention using content qualitative approach to describe participants’ experiences with HoSIP.

**Discussion:**

Through this study we will examine the feasibility of delivering informal home care services to community-dwelling lonely older adults in a developing country through employing a concurrent nested mixed-method design.

**Trial registration:**

IRCT20190503043455N

## Introduction

Aging is accompanied by an increase in the prevalence rate of chronic diseases and functional disabilities [1]. However, compared to past generations, today’s older adults have better functional ability [2]. The majority of older people prefer to age in place, remaining autonomous, active and independent in their own home and surrounded by family and friends [3]. On the other hand, some older people do not have a large social network, and loneliness is one of the most challenging issues that makes living at home hard for older people [4]. The prevalence of loneliness increases among older adults; Jylhä noted that age may not conclusively be a causal factor for feeling lonely, however increasing disability and decreasing social integration with increasing age may be a dominant factor [5].

Loneliness is a negative and unpleasant feeling of stress, which may be experienced through a lack of social contacts [6]. Although loneliness is synonymous with social isolation, they are conceptually different [7]. Social isolation is an objective condition of having minimal contact with other people, while loneliness refers to a subjective feeling which is perceived by only an the individual alone [8]. For instance, individuals may be participating in social gatherings, but they may still suffer from feeling of loneliness. An individual may be socially active but feels alone, and conversely, another individual may not feel alone despite being isolated [9]. In other words, loneliness is not related to frequency of contacts with people, but instead to the quality of relationships and meaningful engagement with others [10].

Based on previous studies, approximately one third of older people experience loneliness in later life [11–13]. The rate of loneliness increases with age, particularly in individuals aged 80 and older [14]. Some factors, including death of a spouse, health deterioration, decreased social contacts, hospitalization, and enhanced functional disability may increase risk of loneliness [15, 16], which in itself may be a risk factor for functional disability and reduced social contacts [17], and depression [18] among older adults. Older adults consequently may also experience difficulties to perform activities of daily living independently [19], but would be relying on informal care services in their home [20]. Therefore, the older adults’ home seems to be an ideal location for interventions and short-term and long-term care services provided by health practitioners, health care providers, specialists, and informal caregivers [21]. Although there are centers that provide home health care services for older adults, these centers are challenged by financial difficulties and inappropriate infrastructure [22, 23].

To provide older adults with informal home care services, comprehensive strategies could be applied to provide a platform for participation of all stakeholders. The use of appropriate technology such as online social networks can support the implementation of these strategies [24]. Maintaining meaningful social relationships is considered an important elements of healthy aging [25]. The geographical distance from relatives and/or functional disabilities may prevent older adults from having social contact, which may result in increased loneliness [26]. Therefore, the use of social media may help older individuals in communicating with each other regardless of geographical location and time [26, 27]. That is "*Social media are Internet-based channels that allow users to opportunistically interact and selectively self-present*, *either in real-time or asynchronously*, *with both broad and narrow audiences who derive value from user-generated content and the perception of interaction with others* [28]".

Social media may provide opportunities for creating social connections, giving and receiving social support, and enhancing a sense of control over life [29]. Receiving emotional support through an online social network could lead to improving functional outcomes among older people [29]. Communication and having relationships with other older adults and friends through online social networks might increase social cohesion and promote social networks. In the context of such social networks, older people can provide each other with emotional, instrumental, communicative and informational support [30]. Stevens et al. found that telephone calls and home visits by peer volunteers, who had similar characteristics, could improve physical activity in older adults [31]. In fact, communication with peer group members can be an effective way to reduce loneliness, disappointment, and social isolation among lonely older adults, and consequently decreasing psychological distress and mortality among older people [21]. Volunteer peers can also play a crucial role in providing peer support and community-based social support. Older adults are likewise very committed to provide peer support as volunteers as they spend more time on community-based services, compared to other age groups [22].

According to the helper therapy principal (HTP), in the context of peer support, individuals receive mutual benefit during contact with peers [32]. The HTP describes that when an older adult provides a specific service for other older adults, the person providing supports also receives benefits, including information exchange, emotional benefit, and increased level of social contacts [33]. In such an environment, older adults are able to share their opinions and perspectives peaceful environment without worry or fear [34]. This approach has reciprocal advantages including improved self-efficacy by helping others and increased self-knowledge and skills because of shared common experiences [32, 35].

In Iran, as a developing country, studies on the provision of the informal supportive home care for older adults are in their infancy. Current evidence on home care services for older adults are limited to some qualitative studies on exploring the barriers of home care programs [23], the role of family support in home care [36], and the necessity of providing health home care services [22]. So, there is a need to investigate the feasibility of interventions that focus on informal home care programs provision involving volunteer peer supports as a cost-effective and mutual beneficial program for those involved.

### Conceptual framework

In the present study, the conceptual framework of peer support, originating from the social support theory, will be used to develop a home care program for older adults [37]. This framework is based on an online social network, and consists of four components including emotional, informational, instrumental, and affiliational support [37, 38]. Emotional support (lonely older adults provide empathy, care, counsel for their peers, and help each other) may build self-confidence and self-esteem [38]; Informational support is the process of knowledge transfer such as health information, educational assistance, learning new skills (vocational or art courses), and learning about their right by peers [38]; Instrumental support comprises the support of peers in performing instrumental activities of daily living (e.g. shopping, cooking, using transportation) [39].; and Affiliational support includes the support of peers in making social relationship between a group of older adults with similar characteristics (living alone), reinforcing the sense of belonging [38].

**Fig 1:**
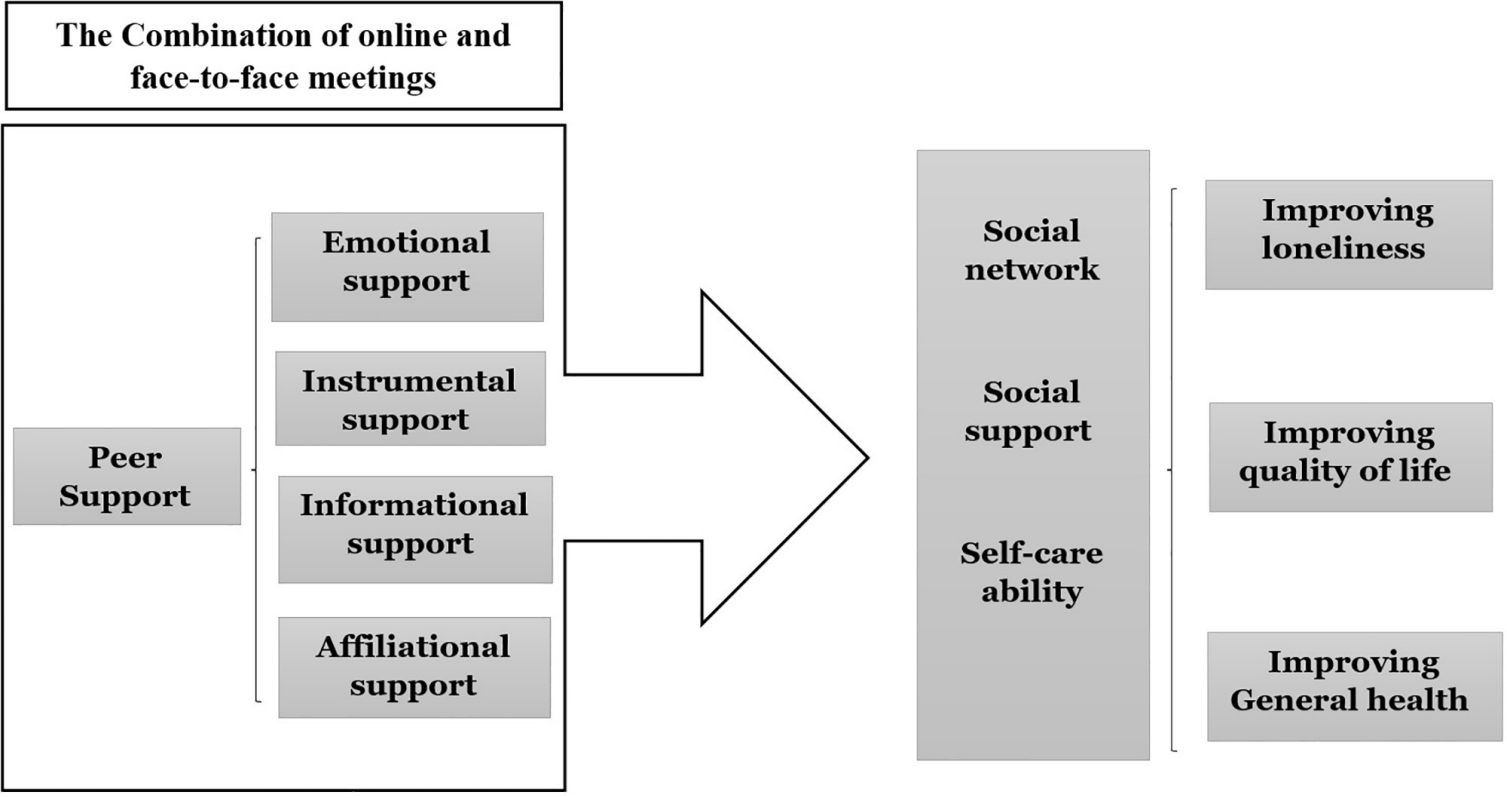
The conceptual framework designed based on social support model.

In fact, peer support could have mutual benefits for both older adults and peer helpers. Our aim is to investigate, if solitary older adults receive the mentioned supports from their older adult peers and how it affects their social network, social support, and self-care ability (Fig 1).

The purpose of this study is to investigate the feasibility of design, implementation, and evaluation of the informal Home care Support Intervention for Loneliness (HoSIP) program for community-dwelling lonely older adults. When compared to their counterparts who do not receive this informal services. In fact, participants will perform for each other four types of supports including emotional, instrumental, informational, and affiliational in the context of online and face-to-face meetings. The first hypothesis is that participants in the intervention group will report lower levels of loneliness compared to those in the control group. The second hypothesis is that compared to the control group, participants in the intervention group will report better levels of health and psychosocial outcomes.

### Study objectives

In this feasibility study, our specific aims, evaluated using Orsmond’s feasibility checklist [40], include:

1. Evaluation of recruitment capability and resulting sample characteristics
2. Evaluation of refinement of data collection procedure and outcome measures
3. Evaluation of the acceptability and suitability of the intervention and study procedures
4. Evaluation of the resource and ability to implement the study and intervention
5. Preliminary evaluation of participant response to intervention

## Materials and methods

This study is approved by the Research Ethics Committee in Tabriz University of Medical Sciences (IR.TBZMED.REC.1399.488). We use a mixed-methods study with concurrent nested design (quantitative and qualitative research methods), HoSIP will be conducted to provide lonely older adults (intervention group) with a 12-week home care service by peer supporters. Quantitative data will be obtained from a cross-sectional study and a pretest-posttest control group intervention study (the pretest will be conducted at the baseline, and the posttest will be performed immediately, three and six months after the intervention period). Qualitative data aiming to identify the feeling of loneliness, quality of life, and general health among participants, as well as strengths and weaknesses of the intervention program, will be collected immediately after the intervention. All participants will complete a consent form before entry into the study. This protocol for a feasibility study was provided based on the SPRIT-13 checklist (S1 and S2 Files).

### Eligibility

Community-dwelling single-living older adults and registered with Iranian national government Electronic Health Records (EHRs) at Gorgan Health Center, North-east of Iran, will be recruited.

### Inclusion criteria

Inclusion criteria for both giver and receiver peers are:

1. Aged 60 years and older living in the same from local neighborhoods
2. Able and willing to provide written informed consent
3. Without any cognitive disability based on Abbreviated Mental Test (AMT_10)
4. Not receiving any personal or practical assistance from others
5. Older adults who live alone at home

### Exclusion criteria

Exclusion criteria includes:

1. Being reluctant to continue study procedures.
2. People with visual and hearing impairments will be referred to related support centers, in the case of not being supported by the Iranian welfare organization.
3. Participating in another healthcare intervention during the previous 12 months

## Phase 1: Cross-sectional study participants

A total of two-hundred and twenty-two community-dwelling lonely older people covered by Gorgan health care-centers with inclusion criteria will be recruited, based on census sampling method. The research team will make contact to community-dwelling older adults (aged ≥ 60 years) registered as lonely persons in the health records in the Health Center of Gorgan city, Iran and explain the purpose of conducting the survey and, invite them to participate in the project. The researcher will suggest an appropriate time and date for a face-to face visit at a day care center (Kanon Salmandi Jahan-Didegan) in Gorgan city, where the questionnaires will be completed under the rules of Covid-19 health protocols (following social distancing of at least two meters, wearing a mask).

### Measures

#### Socio-demographic characteristics and health conditions

Socio-demographic characteristics will be assessed using the following 13 items: age, gender, level of education (illiterate, primary education, secondary education and tertiary education), income (Iranian currency), employment condition (employer, pensioner (somebody who receives a steady salary after her retired husband or father’s death), retired person (an older person who receives a steady salary after finishing his/her own work activity), and homemaker, and others, number of children, living neighborhood (residency) of offspring(s) (15–45 minute buffer zone distance between offspring (s) and older parents), health condition (disability, chronic disease (yes/no), participation in social and religious activities (yes/no), social distress (family conflict (yes/ no), death of close family member and/or friend in the last 6 months (yes/ no), sleep quality (good/ bad), being a daily smoker (yes/no).

### Questionnaires

*Loneliness*. Will be assessed using the Persian version [41], of the 20-item UCLA Loneliness Scale (UCLA -20, Version 3). The range score for this scale is 20–80, and a higher score indicates a more intense level of feeling of loneliness [42]. Cronbach’s alpha coefficient of this questionnaire among Iranian older adults is reported to be 0.81 [41].

*Quality of life*. Will be determined using the 19-item Control, Autonomy, Pleasure and Self-realization (CASP-19). The minimum score of the questionnaire is 0 and the maximum score is 57 [43]. Its internal consistency in the Persian version among Iranian older adults was 0.97 [44].

*General health*. Will be evaluated using 12 item General Health Questionnaire (GHQ12). The total scores ranges from 0 to 36, where higher scores indicate higher level of mental distress [45]. The reliability and validity of this questionnaire among Iranian older adults has been conducted Namjoo and et al. [46].

*Social support*. Will be appraised using the 12 item Multidimensional Scale of Perceived Social Support (MSPSS); possible scores range from 12 to 60. Perceived Social Support scoring comprising low (12–20), moderate (20–40), and high (40+) [47]. The Cronbach’s coefficient alpha value among Iranian older adults was 0.73 [48].

*Self-care ability*. Will be measured using 17-item Self-care Ability Scale for the Elderly (SASE). The minimum score of the questionnaire is 17 and the maximum score is 85 [49]. Its internal consistency in the Persian version among Iranian older adults was 0.73 [50].

*Social network*. Will be assessed using Lubben Support Network Scale-6 (LSNS-6). Total scores is ranged from 0 (low level of social support) to 30 (high level of social support) [51]. The Cronbach’s coefficient alpha among Iranian older people was 0.77 [52].

Moreover, three open-ended questions will be asked to identify support services required at home as well as all the problems related to living alone:

1. What are the challenges with living alone?
2. What services (Personal /Practical) do you need at home?
3. What skills and/or abilities do you have to help your peers? (For both givers and receivers).

## Phase 2: Intervention program

### Requirement and eligibility

Lonely older adults in the cross-sectional study who consent to the second phase of study will evaluate for inclusion in the intervention study. Older adults living alone may participate in some/all the phases of the study (cross-sectional, intervention, and evaluation). Following the cross-sectional baseline assessment, a sample of thirty-two lonely older adults who meet the inclusion criteria will be recruited to the intervention program. The sample size was calculated using the following formula:

$$n=\frac{{\left(Z_{1-\frac{\alpha }{2}}+Z_{1-\beta }\right)}^{2}\left(S_{1}^{2}+S_{2}^{2}\right)}{d^{2}}$$

With 95% confidence interval and 80% power [53]. The sample size is calculated to be 27. Considering a potential attrition rate of 15%, the final sample size is calculated to be 32.

Then, participants in the intervention group will be invited for a an information session held at the day care center, during which all details related to intervention program will be explained. This session will provide an opportunity for intervention group to get to know each other. Participants willing to join the study will have to complete a written informed consent form. Participants will receive informal home care support intervention for loneliness (HoSIP) program through mutual peer support for approximately 12 weeks (3 months).

The participants in the control group with similar characteristic to those of intervention group will be selected from one of the cities of Golestan province, Aliabad-e- Katul, which is 42 Km away from Gorgan city, with an area of 1163 square kilometers. It is the third largest city of Golestan province (Gorgan, Gonbad-Kabus, and Aliabad-e-Katul). Aliabad-e-Katul has similar cultural characrtistics to Gorgan city for instance, different ethnicities such as Fars, Sistani, Kurd, Turk, and Shahrodi all living in both cities [54]. Due to ethical consideration, the control group will receive the same intervention program at the end of the study.

### Intervention

**Fig 2:**
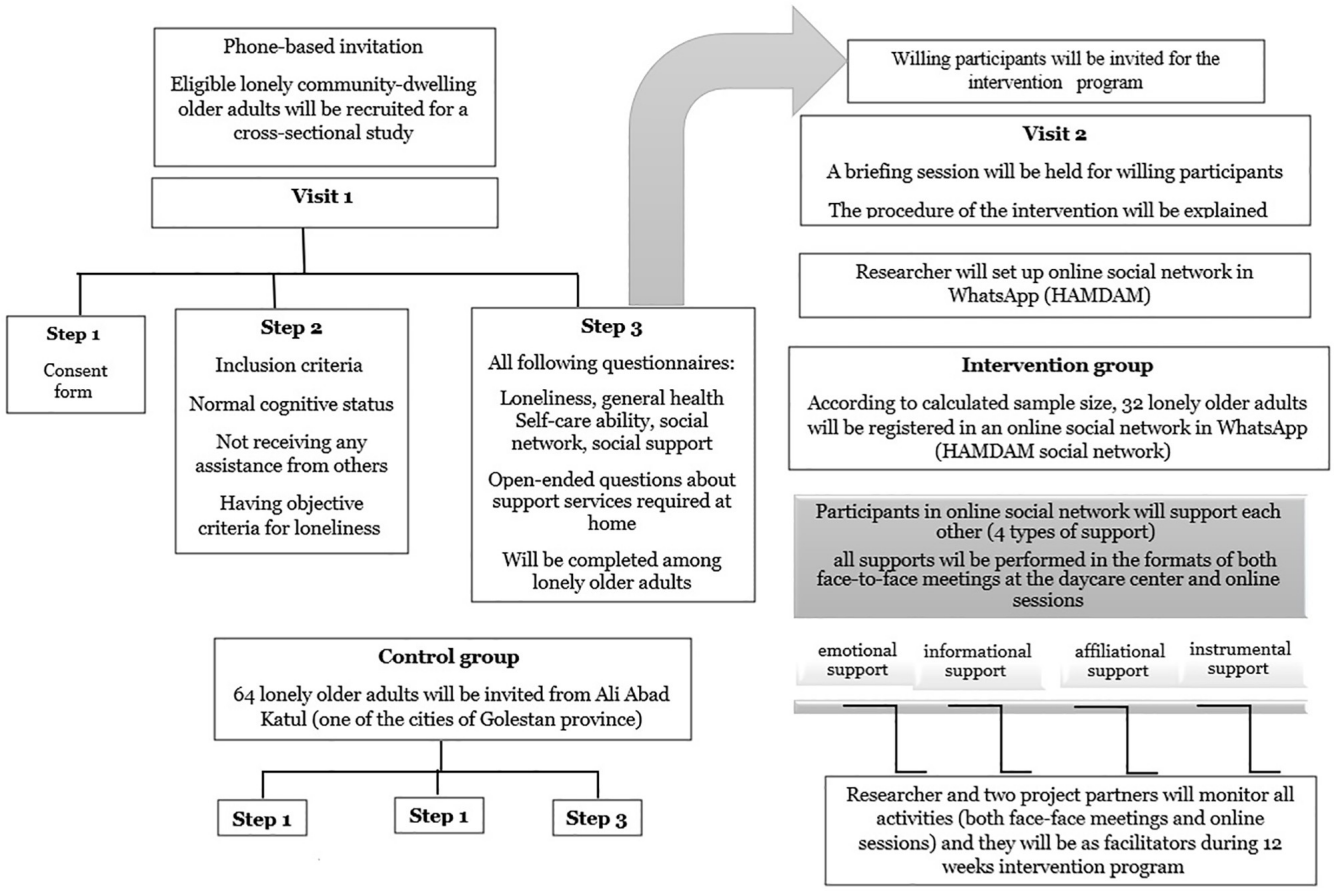
The chart of the intervention phase.

The HoSIP program will provide lonely older adults with informal home care services in the form of scheduled daily activities through an online social networking platform and face-to-face meetings during 12 weeks (3 months). After selecting the participants for the intervention group, the subjects will be registered in an online social network (whatsapp) named "HAMDAM". The participants will be able to express their daily needs such as emotional (e.g. empathy, encouragement), informational (knowledge translation), instrumental (e.g. cooking, shopping), and affiliational (making social relationship between lonely older adults, reinforcing the sense of belonging) supports in the HAMDAM social network consequently, a peer volunteer (lonely older adult) in the HAMDAM social network will provide the desired service for lonely older adults in need of service. In this procedure, all lonely older adults can provide services to any of their peers. For example, if a lonely older adult declares a need in the HAMDAM social network, any member of the group who could meet his/her need can get involved in the informal service delivery. All informal home care services will be carried out in as face-to-face meetings at the daycare center and/or online sessions. These activities will be carried out under the supervision of the first researcher and two project partners. The researcher and two project partners will monitor all activities (both face-face meetings and online sessions) and will be facilitators during 12 weeks (3 months) intervention program (Fig 2).

The phone number of the researcher and two project partners will be given to those older adults who would like to participate in the intervention program but with no online availability. If these lonely older adults need a specific service, they will contact the research team which will see to that a peer volunteer in the network will be contacted by the research team to support the person in need of service. All services will be free of cost and lonely older adults will benefit from mutual support from one to the other in the HAMDAM social network. In the online social network, numerous educational information on healthy aging such as coping with retirement stress, healthy nutrition, self-care, and meaningful activities, will be delivered by the research team once per day to inform lonely older adults about health issues in aging. Fig 2 indicates the procedure of the intervention program.

### Statistical analysis

Data analysis will be conducted using IBM SPSS v26 software. Mean and standard deviation will be reported for continues normally distributed data and interquartile range and median for data deviating from normal distribution. Results of the categorical variables will be presented as percentage and frequency. Independent t-test will be used to determine whether there will be a difference in the feeling of loneliness scores and categorical variables. A chi-square test will be employed to assess a significant association between categorical variables. Regression analysis will be undertaken to explore the relationship between socio-demographic variables and feeling of loneliness. Multiple logistic analysis regression analysis will be conduct to control potential confounding variables including: age, gender, level of education, ethnic, income, and job Analysis of variance with repeated measures will be used to compare outcome measures baseline, post-intervention and follow-up assessments. Paired t-test and covariance analysis will be used to assess the impact of the intervention by adjusting basic comparison and socio-demographic variables in cross-sectional study.

## Phase 3: Qualitative evaluations

### Setting and participants

For further evaluation of the quantitative data, and to address strengths and weaknesses of the intervention program, a qualitative study with conventional content analysis design will be applied using a semi-structured interview among lonely older adults. The purposive sampling will be used to identify lonely older adults having the lowest and the highest scores at the first post-intervention assessment (at the end of 12 weeks) in outcome variables such as feeling of loneliness.

### Data collection

The interviews will be conducted at the day care center. The time of each interview is estimated to be 40–60 minutes and it will be planned by mutual agreement based on participants’ convenience. The participants will be asked to tell about their experiences associated with the intervention program. The semi-structured guide to the interview questions will be asked after the intervention (12 weeks) are as follows:

1. Based on your experiences with the program, how do you assess the services provision in this intervention program?
2. What problems do you identify in this intervention program?
3. What is the strength point of this intervention program?
4. After participation in the program, have you changed your lifestyle?
5. After participation in the program, do you feel any changes in loneliness? How?

As interviews proceed, some probing questions such as "Please, could you elaborate this a little more ", "why?" and "how”, will be asked for exploring the depth of participants’ experience. All interviews will be recorded using a digital recorder and participants’ gestures and facial expressions will be noted during interview.

### Qualitative data analysis

The interviews will be recorded using a digital recorder and transcribed into a text version. Data will be analyzed simultaneously with the collection, immediately after each interview and before starting the next interview. The recorded conversations will be transcribed and managed using MAXQDA 32. The consolidated criteria for reporting qualitative studies checklist (32-items COREQ) will be applied to provide an explicit and comprehensive perspective on semi-structured interviews [55]. Verbatim transcription of interview data will form themes using approach of qualitative content analysis [56]. The member check technique will be applied to receive participants’ approval and feedback on transcripts and it will promote the accuracy, credulity, validity and transferability of this study [57]. The confirmed transcripts will be re-evaluated by two independent researchers to gain the sense of the whole. Then, all sub-themes and themes which have same meaning to all participants will be obtained from meaning unites [58], and the findings will be assessed by two researchers, independently.

### Data storage and protection

All data will be stored in a secure place at the day care center (Kanon Salmandi Jahan-Didegan) in Gorgan city. All interview transcripts will be maintained in secure personal computer system, which will be available only for researchers. Recorded information (audio and visual) will be deleted after transcription into a text version. All data (questionnaires and transcripts) will be kept confidential and anonymous and accessible only for research team members.

### The Integration of the quantitative and qualitative data

**Fig 3:**
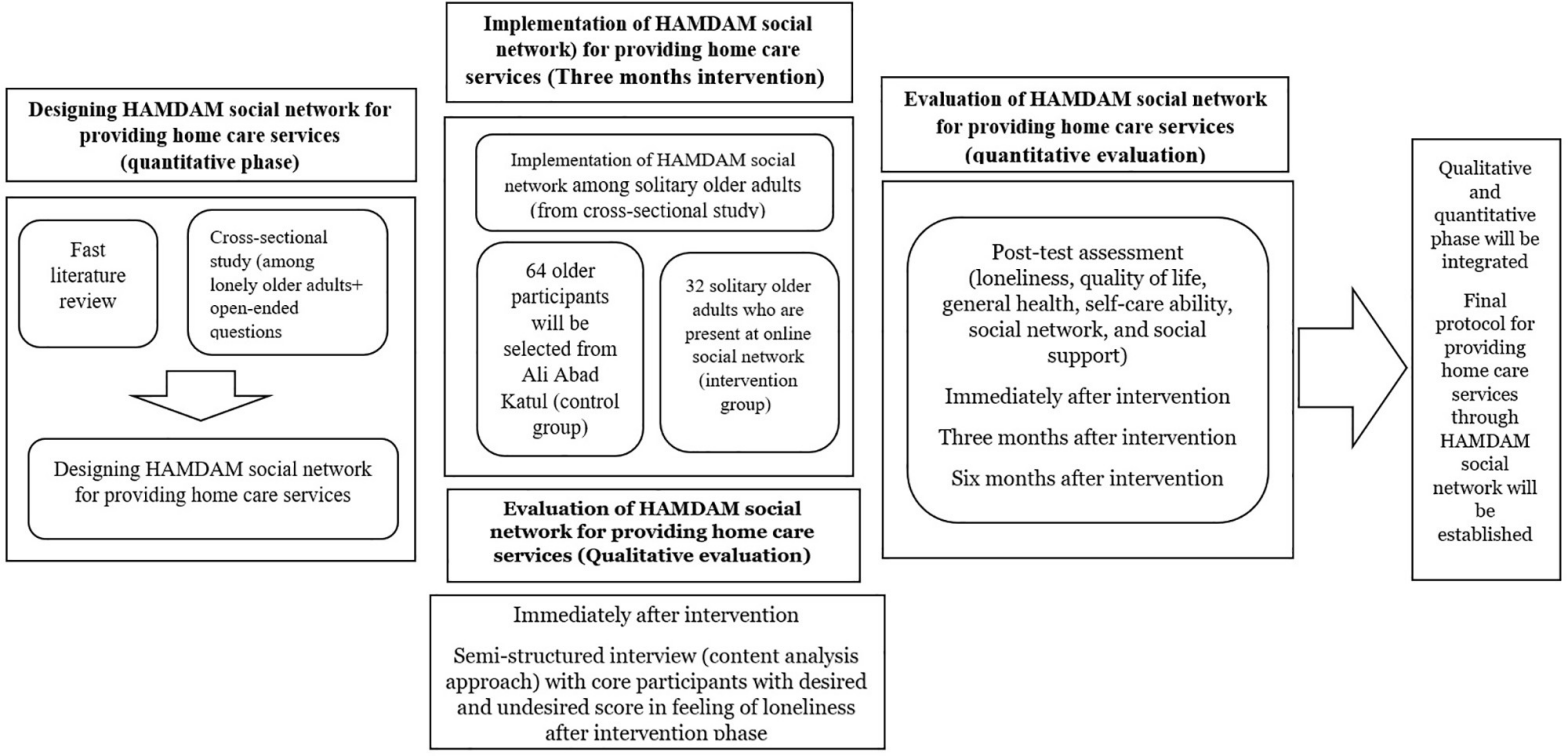
The whole procedure of the study.

To achieve a more comprehensive standpoint about the process of the study, an embedded concurrent design will be conducted. Therefore, quantitative data from the intervention program will be merged with qualitative data from semi-structured interviews. This process will provide us with a total scheme on how much the informal home care service package in the format of combination of face-to-face meetings and online sessions take effect on promoting the primary and secondary outcomes (Fig 3).

### Feasibility outcomes

The following feasibility outcomes (S3 File) will be interpreted based on Orsmond’s feasibility study [40].

1. The main question for the first objective: Can we recruit appropriate participants? This question addresses recruitment rates, eligibility criteria, obstacles to recruitment and the relevance of the intervention to the target population.
2. The main question for the second objective: how appropriate are data collection procedures and outcomes measures for the intended population and for the purpose of the study? This question addresses participants’ ability to complete measures, appropriateness of the data collection, data consistency and usability, appropriateness of the measures to the specific population and intervention.
3. The main question for the third objective: Are study procedures and intervention suitable for and acceptable to participants? This question addresses retention; adherence rates to study procedures, and safety of the procedure in the intervention.
4. The main question for the fourth objective: Dose the research team have the resources and ability to manage the study and intervention? This question addresses the administrative capacity (expertise, skills, and time to conduct the intervention), ethics in implementing the study; budgetary considerations; and technology and equipment needs.
5. The main question for the fifth objective: Does the intervention show promise of being successful with the intended population? This question concentrates on evaluating quantitative and qualitative data of the participants’ responses to the intervention.

### Data monitoring

Monitoring data will be conducted by two supervising professors. They will oversee data precision through a final database assessment following completing data-gathering before conducting statistical analysis. The PhD student will do data collection and oversee checking and informing major unexpected circumstances or unwanted effects of intervention to the research team. This project will be supervised independently by the Research Center on Aging, Tabriz University of Medical Sciences, Tabriz, Iran.

## Discussion

The purpose of the current study is to determine the feasibility and acceptability of implementing an intervention program. The HoSIP to lonely older adults in Gorgan. A novelty of the present study is the use of informal home care services to tackle loneliness among community dwelling older adults, which will be extracted from literature review and cross-sectional study. Moreover, this study will underline the importance of peer supporters for a new way to implement informal home care services. Prior studies have not considered three highly relevant factors, i.e. loneliness, informal home care services, and peer supporters, concomitantly.

Indeed, this feasibility survey will evaluate the relationship between providing informal home care services and socio-psychological consequences among lonely older adults for future extended study. The findings of the present study will have considerable implications for future care and tackling of loneliness among community-dwelling older adults. Furthermore, policy makers will achieve valuable information on the plausible reasons relating to the influence of providing informal home care services among community-dwelling lonely older adults. Therefore, they will be able to make fundamental changes on the current informal home care services for lonely older adults in a developing country like Iran.

### Dissemination

Dissemination will consist of presenting survey’s result to participants, and deliver to the academic society through publishing open-access articles (PhD student’s thesis), and as oral and poster presentations at conferences. The results from this feasibility study will be used to prepare a future extended study. The final assessments and results related to the study will be transferred to all participants.

## Supporting information

S1 File SPIRIT 2013 checklist: Recommended items to address in a clinical trial protocol and related documents*. (DOC)

S2 File Schedule of enrolment, interventions and assessments (SPIRIT). SPIRIT = Standard Protocol Items: Recommendations for Interventional Trials.
(DOCX)

S3 File Objectives and guiding questions for the feasibility study. (DOCX)

S4 File Schedule of enrolment, interventions and assessments (SPIRIT). SPIRIT = Standard Protocol Items: Recommendations for Interventional Trials.
(DOCX)

S5 File Objectives and guiding questions for a feasibility study. (DOCX)

S6 File (DOCX)
