# Supplementary material for: Design, implementation and evaluation of informal home care support intervention program for lonely older adults in the community: Protocol for a feasibility study
Source: PLoS One. 2022 Aug 31;17(8):e0273924. doi: 10.1371/journal.pone.0273924 (PMC9432751; doi:10.1371/journal.pone.0273924)
Supplement: S2 File — SPIRIT = Standard Protocol Items: Recommendations for Interventional Trials. (DOCX) [file pone.0273924.s002.docx]

| **STUDY PERIODE** | | | | | | | | | | | | | | | |  |
| --- | --- | --- | --- | --- | --- | --- | --- | --- | --- | --- | --- | --- | --- | --- | --- | --- |
| **Follow-up after intervention completed** | | | **Intervention period**  **(12 weeks)** | | | | | | | | | | | **Allocation** | **Enrolment** |  |
| T_3_ | T_2_ | T _1_ | 12 | 11 | 10 | 9 | 8 | 7 | 5 | 4 | 3 | 2 | 1 | N/A^*^ | T_0_ | **TIMEPOINT** |
|  |  |  |  |  |  |  |  |  |  |  |  |  |  |  |  | ENROLEMENT: |
|  |  |  |  |  |  |  |  |  |  |  |  |  |  |  | √ | Eligibility screen using  Inclusion/exclusion criteria |
|  |  |  |  |  |  |  |  |  |  |  |  |  |  |  |  |  |
|  |  |  |  |  |  |  |  |  |  |  |  |  |  |  | √ | Informed consent |
|  |  |  |  |  |  |  |  |  |  |  |  |  |  |  | N/A^*^ | Randomization |
|  |  |  |  |  |  |  |  |  |  |  |  |  |  |  |  | INTERVENTION:  **Intervention Group**  Instrumental/ Emotional  Affiliational/ Informational  support  Face to face and online meetings (by peer support approach)  **Control Group**  available care services in the community |
|  |  |  |  |  |  |  |  |  |  |  |  |  |  |  |  |  |
|  |  |  |  |  |  |  |  |  |  |  |  |  |  |  |  |  |
|  |  |  |  |  |  |  |  |  |  |  |  |  |  |  |  |  |
|  |  |  |  |  |  |  |  |  |  |  |  |  |  |  |  | ASSESSMENTS: |
|  |  |  |  |  |  |  |  |  |  |  |  |  |  |  | √ | AMT-10 |
| **√** | **√** | **√** |  |  |  |  |  |  |  |  |  |  |  |  | √ | Demographic |
| **√** | **√** | **√** |  |  |  |  |  |  |  |  |  |  |  |  | √ | UCLA-20 |
| **√** | **√** | **√** |  |  |  |  |  |  |  |  |  |  |  |  | √ | CAPS-19 |
| **√** | **√** | **√** |  |  |  |  |  |  |  |  |  |  |  |  | √ | GHQ-12 |
| **√** | **√** | **√** |  |  |  |  |  |  |  |  |  |  |  |  | √ | MSPSS-12 |
| **√** | **√** | **√** |  |  |  |  |  |  |  |  |  |  |  |  | √ | LSNS-6 |
| **√** | **√** | **√** |  |  |  |  |  |  |  |  |  |  |  |  | √ | SASE-17 |

**Supporting Information File 2.** Schedule of enrolment, interventions and assessments (SPIRIT). SPIRIT = Standard Protocol Items: Recommendations for Interventional Trials

N/A^*^: NOT APPLICABLE; T_1_: immediately after intervention; T_2_: three months after intervention; T_3_: six months after intervention; AMT-10: The Abbreviated Mental Test; UCLA-20: UCLA Loneliness Scale (Version 3); CAPS-19: Control, Autonomy, Pleasure and Self-realization; GHQ-12: General Health Questionnaire; MSPSS-12: Multidimensional Scale of Perceived Social Support; LSNS-6: Lubben Support Network Scale ; SASE-17: Self-care Ability Scale for the Elderly
