# Supplementary material for: Design, implementation and evaluation of informal home care support intervention program for lonely older adults in the community: Protocol for a feasibility study
Source: PLoS One. 2022 Aug 31;17(8):e0273924. doi: 10.1371/journal.pone.0273924 (PMC9432751; doi:10.1371/journal.pone.0273924)
Supplement: S3 File — (DOCX) [file pone.0273924.s003.docx]

**Supporting Information File 3.** Objectives and Guiding Questions for the Feasibility Study

| Objective/ questions | Response | Explanations |
| --- | --- | --- |
| **Objective 1: Evaluation of Recruitment Capability and Resulting Sample Characteristic** |  |  |
| **Main Question: Can we recruit appropriate participants?** |  |  |
| **1. How many potential eligible members of the targeted population are accessible in the local community?** |  |  |
| **2- What are the recruitment rates?** |  |  |
| a. How many participants enter the study at a time? |  |  |
| b. How long does it take to recruit enough participants into the study? |  |  |
| c. What are the refusal rates for participation? |  |  |
| **3. How feasible and suitable are eligibility criteria?** |  |  |
| a. Are criteria clear and sufficient or too inclusive or restrictive? |  |  |
| **4. What are the obstacles to recruitment?** |  |  |
| a. Are colleagues and local organizations willing to assist with recruitment? |  |  |
| b. What are the reasons for refusal or ineligibility? |  |  |
| **5. How relevant is the intervention to the intended population?** |  |  |
| a. Do study participants show evidence of need for the intervention? |  |  |
| c. Are the characteristics of the study participants consistent with the range of expected characteristics as informed by the research literature? |  |  |
| **Objective 2: Evaluation and Refinement of Data Collection Procedures and Outcome Measures**. |  |  |
| **Main Question: How appropriate are the data collection procedures and outcome measures for the intended population and purpose of the study?** |  |  |
| **1. How feasible and suitable are the data collection procedures?** |  |  |
| a. Do participants understand the questions and other data collection procedures? |  |  |
| b. Do they respond with missing or unusable data? |  |  |
| **2. How feasible and suitable is the amount of data collection?** |  |  |
| a. Do the participants have the capacity to complete the data collection procedures? |  |  |
| b. Does the overall data collection plan involve a reasonable amount of time or does it create a burden for the participants? |  |  |
| **3- Do the measures appear to be performing in a consistent way with the intended population as compared to measurement information available in the research literature?** |  |  |
| a. Are internal consistency indicators of measures with the recruited sample congruent with expectations based on prior studies reported in the research literature? |  |  |
| b. Do planned outcome measures appear to be sensitive to the effects of the intervention? |  |  |
| c. Does a suitable outcome measure need to be developed? |  |  |
| **Objective 3: Evaluation of Acceptability and Suitability of Intervention and Study Procedures.** |  |  |
| Main Question: Are study procedures and intervention suitable for and acceptable to participants? |  |  |
| **1. What are the retention and follow-up rates as the participants move through the study and intervention?** |  |  |
| **2. What are the adherence rates to study procedures, intervention attendance, and engagement?** |  |  |
| a. Does the intervention fit with the daily life activities of study participants? |  |  |
| b. Do the participants have enough time and capacity to complete the intervention? |  |  |
| c. Does the intervention involve a reasonable amount of time or does it create a burden for the participants? |  |  |
| d. To what extent is the intervention acceptable and appealing to participants? |  |  |
| e. If appropriate, how many participants agree to be randomized to group? |  |  |
| 3. What is the level of safety of the procedures in the intervention? |  |  |
| a. Are there any unexpected adverse events? |  |  |
| **Objective 4: Evaluation of Resources and Ability to Manage and Implement the Study and Intervention.** |  |  |
| **Main Question: Does the research team have the resources and ability to manage the study and intervention?** |  |  |
| **1. Does the research team have the administrative capacity, expertise, skills, space and time to conduct the study and intervention?** |  |  |
| **2. Can we conduct the study procedures and intervention in an ethical manner?** |  |  |
| a. To what extent does staff comply with the approved human participants’ protocol? |  |  |
| b. How effectively are adverse events during implementation identified, documented, and reported? |  |  |
| **3. Can the study and intervention be conducted within the designated budget?** |  |  |
| **4. Is the technology and equipment sufficient to conduct the study and intervention, including collection, management, and analysis of data?** |  |  |
| a. Is equipment available when needed? |  |  |
| b. What is involved in training personal and/or participants to use the equipment? |  |  |
| 5. Are we able to efficiently and effectively manage data entry and analysis? |  |  |
| Objective 5: Preliminary Evaluation of Participant Responses to Intervention |  |  |
| Main Question: Does the intervention show promise of being successful with the intended population? |  |  |
| **1. Does examination of quantitative data suggest that the intervention is likely to be successful?** |  |  |
| a. Does examination of the data at the participant level suggest that changes in key outcome variables occurred? |  |  |
| b. Are the changes of the outcome variable(s) in the expected direction? |  |  |
| c. Do the estimates of effects suggest that the intervention has promise? |  |  |
| **2. Do participants or relevant others provide qualitative feedback that may be indicative of the likelihood that the intervention will be successful?** |  |  |
| **3. If the quantitative and/or qualitative data suggest that the intervention is not promising:** |  |  |
| a. Are the data collection procedures and outcome measures appropriate for the population and study? |  |  |
| b. Are the outcome measures and intervention theoretically aligned? |  |  |
| c. Is there evidence that the intervention does not produce change in the desired outcomes? |  |  |
| d. Is there evidence that the intervention was not implemented in the intended manner? |  |  |
| e. Have too many adaptations been made in the intervention process to adequately assess the participants’ responses to the intervention? |  |  |
| b. Are the findings congruent with the proposed theoretical model for the intervention? |  |  |

Rference: Orsmond GI, Cohn ES. The distinctive features of a feasibility study: objectives and guiding questions. OTJR: occupation, participation and health. 2015;35(3):169-77.
