# Supplementary material for: Design, implementation and evaluation of informal home care support intervention program for lonely older adults in the community: Protocol for a feasibility study
Source: PLoS One. 2022 Aug 31;17(8):e0273924. doi: 10.1371/journal.pone.0273924 (PMC9432751; doi:10.1371/journal.pone.0273924)
Supplement: S6 File — (DOCX) [file pone.0273924.s006.docx]

**Title: Design, Implementation and Evaluation of Informal Home Care Support Intervention Program for Lonely Older Adults in the Community: 
Protocol for a Feasibility study**

**Introduction**

Aging is accompanied by an increase in the prevalence rate of chronic diseases and functional disabilities(1). However, compared to past generations, today's older adults have better functional ability(2). The majority of older people prefer to age in place, remaining autonomous, active and independent in their own home and surrounded by family and friends (3). On the other hand, some older people do not have a large social network, and loneliness is one of the most challenging issues that makes living at home hard for older people(4). The prevalence of loneliness increases among older adults; Jylhä noted that age may not conclusively be a causal factor for feeling lonely, however increasing disability and decreasing social integration with increasing age may be a dominant factor (5).

Loneliness is a negative and unpleasant feeling of stress, which may be experienced through a lack of social contacts(6). Although loneliness is synonymous with social isolation, they are conceptually different(7). Social isolation is an objective condition of having minimal contact with other people, while loneliness refers to a subjective feeling which is perceived by only an the individual alone(8). For instance, individuals may be participating in social gatherings, but they may still suffer from feeling of loneliness. An individual may be socially active but feels alone, and conversely, another individual may not feel alone despite being isolated(9). In other words, loneliness is not related to frequency of contacts with people, but instead to the quality of relationships and meaningful engagement with others (10).

Based on previous studies, approximately one third of older people experience loneliness in later life (11-13). The rate of loneliness increases with age, particularly in individuals aged 80 and older(14). Some factors, including death of a spouse, health deterioration, decreased social contacts, hospitalization, and enhanced functional disability may increase risk of loneliness(15, 16), which in itself may be a risk factor for functional disability and reduced social contacts (17), and depression (18) among older adults. Older adults consequently may also experience difficulties to perform activities of daily living independently (19), but would be relying on informal care services in their home(20). Therefore, the older adults' home seems to be an ideal location for interventions and short-term and long-term care services provided by health practitioners, health care providers, specialists, and informal caregivers (21). Although there are centers that provide home health care services for older adults, these centers are challenged by financial difficulties and inappropriate infrastructure(22, 23).

To provide older adults with informal home care services, comprehensive strategies could be applied to provide a platform for participation of all stakeholders. The use of appropriate technology such as online social networks can support the implementation of these strategies(24). Maintaining meaningful social relationships is considered an important elements of healthy aging(25). The geographical distance from relatives and/or functional disabilities may prevent older adults from having social contact, which may result in increased loneliness (26). Therefore, the use of social media may help older individuals in communicating with each other regardless of geographical location and time(26, 27). That is "*Social media are Internet-based channels that allow users to opportunistically interact and selectively self-present, either in real-time or asynchronously, with both broad and narrow audiences who derive value from user-generated content and the perception of interaction with others*(28)."

Social media may provide opportunities for creating social connections, giving and receiving social support, and enhancing a sense of control over life(29). Receiving emotional support through an online social network could lead to improving functional outcomes among older people(29). Communication and having relationships with other older adults and friends through online social networks might increase social cohesion and promote social networks. In the context of such social networks, older people can provide each other with emotional, instrumental, communicative and informational support (30). Stevens et al. found that telephone calls and home visits by peer volunteers, who had similar characteristics, could improve physical activity in older adults(31). In fact, communication with peer group members can be an effective way to reduce loneliness, disappointment, and social isolation among lonely older adults, and consequently decreasing psychological distress and mortality among older people(21). Volunteer peers can also play a crucial role in providing peer support and community-based social support. Older adults are likewise very committed to provide peer support as volunteers as they spend more time on community-based services, compared to other age groups (22).

According to the helper therapy principal (HTP), in the context of peer support, individuals receive mutual benefit during contact with peers(32). The HTP describes that when an older adult provides a specific service for other older adults, the person providing supports also receives benefits, including information exchange, emotional benefit, and increased level of social contacts(33). In such an environment, older adults are able to share their opinions and perspectives peaceful environment without worry or fear(34). This approach has reciprocal advantages including improved self-efficacy by helping others and increased self-knowledge and skills because of shared common experiences(32, 35).

In Iran, as a developing country, studies on the provision of the informal supportive home care for older adults are in their infancy. Current evidence on home care services for older adults are limited to some qualitative studies on exploring the barriers of home care programs (23), the role of family support in home care (36), and the necessity of providing health home care services(22). So, there is a need to investigate the feasibility of interventions that focus on informal home care programs provision involving volunteer peer supports as a cost-effective and mutual beneficial program for those involved.

**Conceptual framework**

In the present study, the conceptual framework of peer support, originating from the social support theory, will be used to develop a home care program for older adults(37). This framework is based on an online social network, and consists of four components including emotional, informational, instrumental, and affiliational support (37, 38). Emotional support (lonely older adults provide empathy, care, counsel for their peers, and help each other) may build self-confidence and self-esteem (38); Informational support is the process of knowledge transfer such as health information, educational assistance, learning new skills (vocational or art courses), and learning about their right by peers(38); Instrumental support comprises the support of peers in performing instrumental activities of daily living (e.g. shopping, cooking, using transportation)(39).; and Affiliational support includes the support of peers in making social relationship between a group of older adults with similar characteristics (living alone), reinforcing the sense of belonging(38).

In fact, peer support could have mutual benefits for both older adults and peer helpers. Our aim is to investigate, if solitary older adults receive the mentioned supports from their older adult peers and how it affects their social network, social support, and self-care ability. Fig 1. shows the conceptual framework designed based on social support model.

**
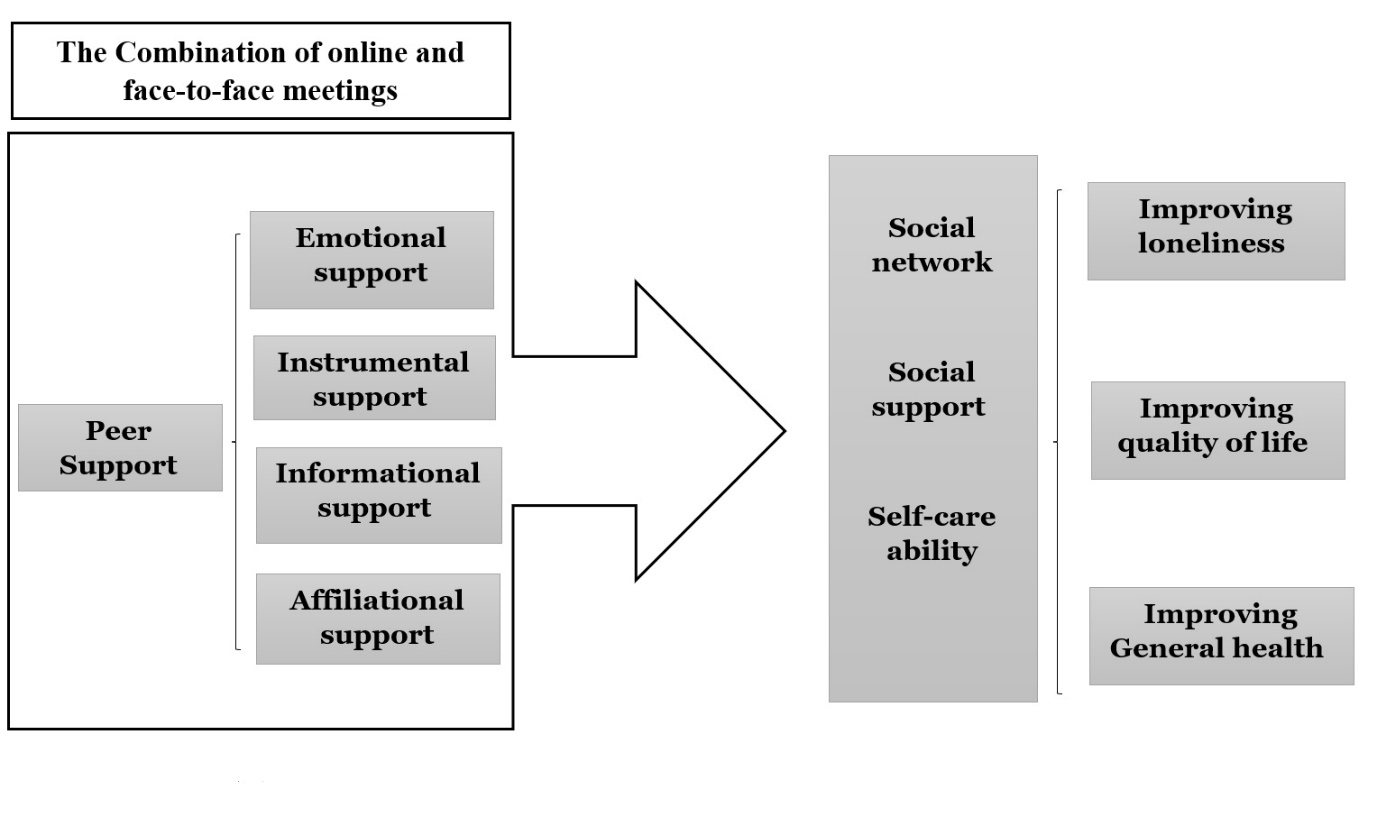
**

The conceptual framework designed based on social support model

**The main objective**

Design, Implementation and evaluation of a Home Care Support Intervention Program for lonely older adults in Gorgan.

**The feasibility of conducting the study**

The implementation process of the current study and the intention of generalizing the results to a larger population will be accompanied by several challenges. The research team will try to tackle these challenges through using feasibility components. Some possible challenges are also pointed out: 1- the reluctance of older adults to participate in the study due to lack of interest in changing their past lifestyle, lack of older adults' knowledge about the benefits of participating in such programs; 2- the possible loss of sample size; 3- lack of researcher's knowledge about older adults' compliances to the intervention program; 4- Safety and ethical issues related to the presence of older adults in each other's house; 5- older adults who will participate in the intervention program may have a higher motivation than those who will refuse to participate; 6- if women older adults participate in this study, the possibility of generalizing the results to a larger population will be questioned; 7- because of cultural issues, provision informal supportive services by lonely older men who don't have family ties may be challenging for lonely older women. Therefore, in order to evaluate the feasibility of home care informal support care services, five criteria related to feasibility study will be evaluated (Objectives and Guiding Questions for a Feasibility Study-supportive file 3).

Objective 1: Evaluation of Recruitment Capability and Resulting Sample Characteristics.

Objective 2: Evaluation and Refinement of Data Collection Procedures and Outcome Measures

Objective 3: Evaluation of Acceptability and Suitability of Intervention and Study Procedures.

Objective 4: Evaluation of Resources and Ability to Manage and Implement the Study and Intervention

Objective 5: Preliminary Evaluation of Participant Responses to Intervention

**The definition of specific words**

**1- Concurrent nested method**

**Theoretical definition**: In this model, both qualitative and quantitative data are being collected at the same time. Qualitative data are embedded in the quantitative design(40).

**Practical definition:** in this study quantitative questionnaires and semi-structured interview will be conducted simultaneously to evaluate the intervention program.

2- **Home care**

**Theoretical definition**: Home care services are suitable for older adults who would like to live in their homes until the end of their lives. These services are provided in two ways: personal care and informal help services(41).

**Practical definition**: in this study, lonely older adults will perform some informal support (instrumental, informational, affilitional and emotional) for each other at home and in community.

**3- aging**

**Theoretical definition**: Older adults who are at the age of 60 and over and they experience changes in different aspects, i.e. physical, psychological and social (42).

**Practical definition**: in this study we will consider older adults at the age of 60 and over

**4- peer support**

**Theoretical definition**: peer supporters are people who have the same characteristic as person who is in need of help. Peer supporters usually provide each other emotional and social support in the community(43).

**Practical definition**: In this study, lonely older adults will provide each other instrumental, informational, affilitional and emotional support.

**5- online social network**

**Theoretical definition**: An online service or site to facilitate social interaction to help individuals find others of a common interest, establish a forum for discussion, and exchange information(44).

**Practical definition**: in this study lonely older adult will be registered in online social network (HAMDAM).

**The ethical considerations**

1- This study is approved by the Research Ethics Committee in Tabriz University of Medical Sciences (IR.TBZMED.REC.1399.488)

2- Verbal and written informed consent from all participants will be obtained.

3- All participants will be informed about using a voice recorder and taking notes from the interviews

4- The time and the place of the assessment (completing questionnaires and interviews) will be chosen by participants

5- The identities of all participants will remain confidential

6- All publications resulting from this thesis should be published under the name of Tabriz University of Medical Sciences.

**Eligibility**

**Inclusion criteria**

Inclusion criteria for both giver and receiver peers are:

1. Aged 60 years and older living in the same from local neighborhoods
2. Able and willing to provide written informed consent
3. Without any cognitive disability based on Abbreviated Mental Test (AMT_10)
4. Not receiving any personal or practical assistance from others
5. Older adults who live alone at home

**Exclusion criteria**

Exclusion criteria includes:

1. Being reluctant to continue study procedures.
2. People with visual and hearing impairments will be referred to related support centers, in the case of not being supported by the Iranian welfare organization.
3. Participating in another healthcare intervention during the previous 12 months

**Method/design:**

We will use a mixed-methods study with concurrent nested design (quantitative and qualitative research methods), HoSIP will be conducted to provide solitary older adults (intervention group) with a 12-week home care service by peer supporters. Quantitative data will be obtained from a cross-sectional study and a pretest-posttest control group intervention study (the pretest will be conducted at the baseline, and the posttest will be performed immediately, three and six months after the intervention period). Qualitative data aiming to identify the feeling of loneliness, quality of life, and general health among participants, as well as strengths and weaknesses of the intervention program, will be collected immediately after the intervention. All participants will complete a consent form before entry into the study. This study will be conducted in three phases including two quantitative stages and one quantitative (qualitative) phase

**Quantitative phases**

**Phase 1: Cross-sectional study**

**The type of study:** We will conduct a descriptive and analytical cross-sectional study.

**Study population:** Community-dwelling single-living older adults and registered with Iranian national government Electronic Health Records (EHRs) at Gorgan Health Center, North-east of Iran, will be recruited.

**The method of collecting information**

A total of two-hundred and twenty-two community-dwelling lonely older people covered by Gorgan health care-centers with inclusion criteria will be recruited, based on census sampling method. The research team will make contact to community-dwelling older adults (aged ≥ 60 years) registered as lonely persons in the health records in the Health Center of Gorgan city, Iran and explain the purpose of conducting the survey and, invite them to participate in the project. The researcher will suggest an appropriate time and date for a face-to face visit at a day care center (Kanon Salmandi Jahan-Didegan) in Gorgan city, where the questionnaires will be completed under the rules of Covid-19 health protocols (following social distancing of at least two meters, wearing a mask).

**Measures**

**Socio-demographic characteristics and health conditions**

Socio-demographic characteristics will be assessed using the following 13 items: age, gender, level of education (illiterate, primary education, secondary education and tertiary education), income (Iranian currency), employment condition (employer, pensioner (somebody who receives a steady salary after her retired husband or father’s death) , retired person (an older person who receives a steady salary after finishing his/her own work activity ), and homemaker, and others, number of children, living neighborhood (residency) of offspring(s) ( 15-45 minute buffer zone distance between offspring (s) and older parents), health condition (disability, chronic disease (yes/no), participation in social and religious activities (yes/no), social distress (family conflict (yes/ no), death of close family member and/or friend in the last 6 months (yes/ no), sleep quality (good/ bad ), being a daily smoker (yes/no).

**Questionnaires**

**Loneliness** will be assessed using the Persian version(45), of the 20-item UCLA Loneliness Scale (UCLA -20, Version 3). The range score for this scale is 20-80, and a higher score indicates a more intense level of feeling of loneliness(46). Cronbach's alpha coefficient of this questionnaire among Iranian older adults is reported to be 0.81 (45).

**Quality of life** will be determined using the 19-item Control, Autonomy, Pleasure and Self-realization (CASP-19). The minimum score of the questionnaire is 0 and the maximum score is 57 (47). Its internal consistency in the Persian version among Iranian older adults was 0.97 (48).

**General health** will be evaluated using 12 item General Health Questionnaire (GHQ12). The total scores ranges from 0 to 36, where higher scores indicate higher level of mental distress(49). The reliability and validity of this questionnaire among Iranian older adults has been conducted Namjoo and et.al (50).

**Social support** will be appraised using the 12 item Multidimensional Scale of Perceived Social Support (MSPSS); possible scores range from 12 to 60. Perceived Social Support scoring comprising low (12-20), moderate (20-40), and high (40+)(51). The Cronbach's coefficient alpha value among Iranian older adults was 0.73 (52)

**Self-care ability** will be measured using 17-item Self-care Ability Scale for the Elderly (SASE). The minimum score of the questionnaire is 17 and the maximum score is 85(53). Its internal consistency in the Persian version among Iranian older adults was 0.73 (54).

**Social network** will be assessed using Lubben Support Network Scale-6 (LSNS-6). Total scores is ranged from 0 (low level of social support) to 30 (high level of social support)(55). The Cronbach's coefficient alpha among Iranian older people was 0.77(56).

Moreover, three open-ended questions will be asked to identify support services required at home as well as all the problems related to living alone:

1. What are the challenges with living alone?
2. What services (Personal /Practical) do you need at home?
3. What skills and/or abilities do you have to help your peers? (For both givers and receivers).

**Statistical analysis**

Data analysis will be conducted using IBM SPSS v26 software. Mean and standard deviation will be reported for continues normally distributed data and interquartile range and median for data deviating from normal distribution. Results of the categorical variables will be presented as percentage and frequency. Independent t-test will be used to determine whether there will be a difference in the feeling of loneliness scores and categorical variables. A chi-square test will be employed to assess a significant association between categorical variables. Regression analysis will be undertaken to explore the relationship between socio-demographic variables and feeling of loneliness. Multiple logistic analysis regression analysis will be conduct to control potential confounding variables including: age, gender, level of education, ethnic, income, and job.

**Phase 2: intervention program**

**Type of the study:** This is a quasi-experimental study, pre-post intervention, with control group.

**Sample size:** Following the cross-sectional baseline assessment, a sample of thirty-two lonely older adults who meet the inclusion criteria will be recruited to the intervention program. The sample size was calculated using the following formula:


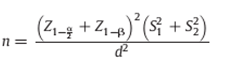


With 95% confidence interval and 80% power(57). The sample size is calculated to be 27. Considering a potential attrition rate of 15%, the final sample size is calculated to be 32.

**The method of collecting information**

Then, participants in the intervention group will be invited for an information session held at the day care center, during which all details related to intervention program will be explained. This session will provide an opportunity for intervention group to get to know each other. Participants willing to join the study will have to complete a written informed consent form. Participants will receive informal home care support intervention for loneliness (HoSIP) program through mutual peer support for approximately 12 weeks (3 months).

The participants in the control group with similar characteristic to those of intervention group will be selected from one of the cities of Golestan province, Aliabad-e- Katul, which is 42 Km away from Gorgan city, with an area of 1163 square kilometers. It is the third largest city of Golestan province (Gorgan, Gonbad-Kabus, and Aliabad-e-Katul). Aliabad-e-Katul has similar cultural characrtistics to Gorgan city for instance, different ethnicities such as Fars, Sistani, Kurd, Turk, and Shahrodi all living in both cities (58). Due to ethical consideration, the control group will receive the same intervention program at the end of the study.

The HoSIP program will provide lonely older adults with informal home care services in the form of scheduled daily activities through an online social networking platform and face-to-face meetings during 12 weeks (3 months). After selecting the participants for the intervention group, the subjects will be registered in an online social network (whatsapp) named "HAMDAM". The participants will be able to express their daily needs such as emotional (e.g. empathy, encouragement), informational (knowledge translation), instrumental (e.g. cooking, shopping), and affiliational (making social relationship between lonely older adults, reinforcing the sense of belonging) supports in the HAMDAM social network consequently, a peer volunteer (lonely older adult) in the HAMDAM social network will provide the desired service for lonely older adults in need of service. In this procedure, all lonely older adults can provide services to any of their peers. For example, if a lonely older adult declares a need in the HAMDAM social network, any member of the group who could meet his/her need can get involved in the informal service delivery. All informal home care services will be carried out in as face-to-face meetings at the daycare center and/or online sessions. These activities will be carried out under the supervision of the first researcher and two project partners. The researcher and two project partners will monitor all activities (both face-face meetings and online sessions) and will be facilitators during 12 weeks (3 months) intervention program**.**

The phone number of the researcher and two project partners will be given to those older adults who would like to participate in the intervention program but with no online availability. If these lonely older adults need a specific service, they will contact the research team which will see to that a peer volunteer in the network will be contacted by the research team to support the person in need of service. All services will be free of cost and lonely older adults will benefit from mutual support from one to the other in the HAMDAM social network. In the online social network, numerous educational information on healthy aging such as coping with retirement stress, healthy nutrition, self-care, and meaningful activities, will be delivered by the research team once per day to inform lonely older adults about health issues in aging.

**Phase 3: Qualitative evaluations**

**This phase will be conducted in two stages**

**Stage 1:**

post-test will be conducted at three points (immediately after intervention, three months and six months after intervention). All questionnaires (UCLA-20; CAPS-19; GHQ-12; MSPSS-12; LSNS-6; SASE-17) will be completed among intervention group and control group.

**Statistical analysis**

Analysis of variance with repeated measures will be used to compare outcome measures baseline, post-intervention and follow-up assessments. Paired t-test and covariance analysis will be used to assess the impact of the intervention by adjusting basic comparison and socio-demographic variables in cross-sectional study.

**Stage 2:**

**Type of the study:** A qualitative study with content analysis approach will be conducted

**Participants:** The purposive sampling will be used to identify lonely older adults having the lowest and the highest scores at the first post-intervention assessment (at the end of 12 weeks) in outcome variables such as feeling of loneliness.

**Research question:** Based on the participants' experiences, what are the strengths and weaknesses of establishing a social network (HAMDAM) to provide an Informal Home Care Support?

**Data collection**

The interviews will be conducted at the day care center. The time of each interview is estimated to be 40-60 minutes and it will be planned by mutual agreement based on participants' convenience. The participants will be asked to tell about their experiences associated with the intervention program. The semi-structured guide to the interview questions will be asked after the intervention (12 weeks) are as follows:

1- Based on your experiences with the program, how do you assess the services provision in this intervention program?

2- What problems do you identify in this intervention program?

3- What is the strength point of this intervention program?

4- After participation in the program, have you changed your lifestyle?

5- After participation in the program, do you feel any changes in loneliness? How?

As interviews proceed, some probing questions such as "Please, could you elaborate this a little more ", "why?" and "how”, will be asked for exploring the depth of participants' experience. All interviews will be recorded using a digital recorder and participants' gestures and facial expressions will be noted during interview

**Qualitative data analysis**

The interviews will be recorded using a digital recorder and transcribed into a text version. Data will be analyzed simultaneously with the collection, immediately after each interview and before starting the next interview. The recorded conversations will be transcribed and managed using MAXQDA 32. The consolidated criteria for reporting qualitative studies checklist (32-items COREQ) will be applied to provide an explicit and comprehensive perspective on semi-structured interviews (59). Verbatim transcription of interview data will form themes using approach of qualitative content analysis(60). The member check technique will be applied to receive participants' approval and feedback on transcripts and it will promote the accuracy, credulity, validity and transferability of this study (61). The confirmed transcripts will be re-evaluated by two independent researchers to gain the sense of the whole. Then, all sub-themes and themes which have same meaning to all participants will be obtained from meaning unites (62), and the findings will be assessed by two researchers, independently.

**The Integration of the quantitative and qualitative data**

To achieve a more comprehensive standpoint about the process of the study, an embedded concurrent design will be conducted. Therefore, quantitative data from the intervention program will be merged with qualitative data from semi-structured interviews. This process will provide us with a total scheme on how much the informal home care service package in the format of combination of face-to-face meetings and online sessions take effect on promoting the primary and secondary outcomes**.** Fig 2 shows the whole procedure of the study.

**
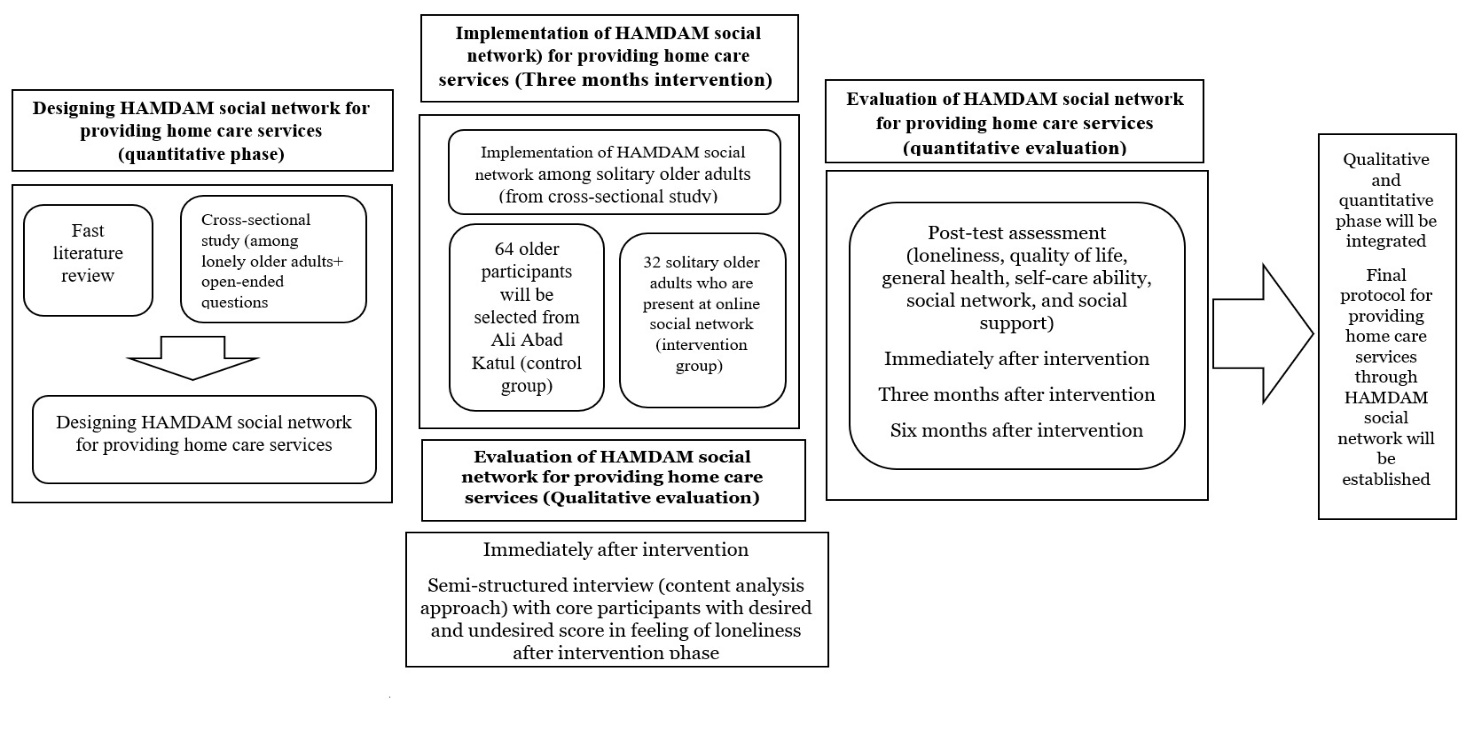
**

Fig 2. The whole procedure of the study.

**References**

1. Freedman VA, Martin LG, Schoeni RF. Recent trends in disability and functioning among older adults in the United States: a systematic review. Jama. 2002;288(24):3137-46.

2. Iecovich E. Aging in place: From theory to practice. Anthropological notebooks. 2014;20(1):21-33.

3. Wiles JL, Leibing A, Guberman N, Reeve J, Allen RE. The meaning of “aging in place” to older people. The gerontologist. 2012;52(3):357-66.

4. Bandari R, Khankeh HR, Shahboulaghi FM, Ebadi A, Keshtkar AA, Montazeri A. Defining loneliness in older adults: protocol for a systematic review. Systematic reviews. 2019;8(1):1-6.

5. Jylhä M. Old age and loneliness: cross-sectional and longitudinal analyses in the Tampere Longitudinal Study on Aging. Canadian Journal on Aging/La revue canadienne du vieillissement. 2004;23(2):157-68.

6. Tiwari SC. Loneliness: A disease? Indian journal of psychiatry. 2013;55(4):320.

7. Courtin E, Knapp M. Social isolation, loneliness and health in old age: a scoping review. Health & social care in the community. 2017;25(3):799-812.

8. Grenade L, Boldy D. Social isolation and loneliness among older people: issues and future challenges in community and residential settings. Australian Health Review. 2008;32(3):468-78.

9. Duncan D, Bell R. Local action on health inequalities: Reducing social isolation across the lifecourse–practice resource 2015 [Available from: <https://assets.publishing.service.gov.uk/government/uploads/system/uploads/attachment_data/file/461120/3a_Social_isolation-Full-revised.pdf>.

10. Mann F, Bone JK, Lloyd-Evans B, Frerichs J, Pinfold V, Ma R, et al. A life less lonely: the state of the art in interventions to reduce loneliness in people with mental health problems. Social psychiatry and psychiatric epidemiology. 2017;52(6):627-38.

11. Hauge S, Kirkevold M. Older Norwegians’ understanding of loneliness. International Journal of Qualitative Studies on Health and Well-being. 2010;5(1):4654.

12. Yan Z, Yang X, Wang L, Zhao Y, Yu L. Social change and birth cohort increase in loneliness among Chinese older adults: a cross-temporal meta-analysis, 1995–2011. International psychogeriatrics. 2014;26(11):1773-81.

13. Ayalon L, Shiovitz-Ezra S. The relationship between loneliness and passive death wishes in the second half of life. International Psychogeriatrics. 2011;23(10):1677-85.

14. Dykstra PA. Older adult loneliness: myths and realities. European journal of ageing. 2009;6(2):91.

15. Squires SE. To a deeper understanding of loneliness amongst older Irish adults. Collegium antropologicum. 2015;39(2):289-95.

16. Valtorta N, Hanratty B. Loneliness, isolation and the health of older adults: do we need a new research agenda? Journal of the Royal Society of Medicine. 2012;105(12):518-22.

17. Niedzwiedz CL, Richardson EA, Tunstall H, Shortt NK, Mitchell RJ, Pearce JR. The relationship between wealth and loneliness among older people across Europe: Is social participation protective? Preventive medicine. 2016;91:24-31.

18. Erzen E, Çikrikci Ö. The effect of loneliness on depression: A meta-analysis. International Journal of Social Psychiatry. 2018;64(5):427-35.

19. Dubuc N, Dubois M-F, Raîche M, Hébert R. Meeting the home-care needs of disabled older persons living in the community: does integrated services delivery make a difference? BMC geriatrics. 2011;11(1):1-13.

20. Dupuis-Blanchard S, Gould ON, Gibbons C, Simard M, Éthier S, Villalon L. Strategies for aging in place: the experience of language-minority seniors with loss of independence. Global qualitative nursing research. 2015;2:2333393614565187.

21. Dyck I, Kontos P, Angus J, McKeever P. The home as a site for long-term care: meanings and management of bodies and spaces. Health & place. 2005;11(2):173-85.

22. Nikbakht-Nasrabadi A, Shabany-Hamedan M. Providing healthcare services at home-a necessity in Iran: a narrative review article. Iranian Journal of Public Health. 2016;45(7):867.

23. Heydari H, Shahsavari H, Hazini A, Nasrabadi AN. Exploring the barriers of home care services in Iran: A qualitative study. Scientifica. 2016;2016.

24. Willard S, Cremers G, Man YP, van Rossum E, Spreeuwenberg M, de Witte L. Development and testing of an online community care platform for frail older adults in the Netherlands: a user-centred design. BMC geriatrics. 2018;18(1):1-9.

25. Hoglund MW, Sadovsky R, Classie J. Engagement in life activities promotes healthy aging in men. Journal of Men's Health. 2009;6(4):354-65.

26. Leist AK. Social media use of older adults: a mini-review. Gerontology. 2013;59(4):378-84.

27. Hutto CJ, Bell C, Farmer S, Fausset C, Harley L, Nguyen J, et al., editors. Social media gerontology: Understanding social media usage among older adults. Web Intelligence; 2015: IOS Press.

28. Carr CT, Hayes RA. Social media: Defining, developing, and divining. Atlantic journal of communication. 2015;23(1):46-65.

29. Boll F, Brune P. Online support for the elderly–why service and social network platforms should be integrated. Procedia Computer Science. 2016;98:395-400.

30. Schwei RJ, Amesoudji AW, DeYoung K, Madlof J, Zambrano-Morales E, Mahoney J, et al. Older adults’ perspectives regarding peer-to-peer support programs and maintaining independence. Home health care services quarterly. 2020;39(4):197-209.

31. Stevens Z, Barlow C, Iliffe S. Promoting physical activity among older people in primary care using peer mentors. Primary health care research & development. 2015;16(2):201-6.

32. Pagano ME, Post SG, Johnson SM. Alcoholics Anonymous-related helping and the helper therapy principle. Alcoholism Treatment Quarterly. 2011;29(1):23-34.

33. Lepore SJ, Buzaglo JS, Lieberman MA, Golant M, Greener JR, Davey A. Comparing standard versus prosocial internet support groups for patients with breast cancer: a randomized controlled trial of the helper therapy principle. Journal of Clinical Oncology. 2014;32(36):4081.

34. Lowthian JA, Lennox A, Curtis A, Wilson G, Rosewarne C, O’Brien D, et al. HOspitals and patients WoRking in Unity (HOW RU?): telephone peer support to improve older patients’ quality of life after emergency department discharge in Melbourne, Australia—a multicentre prospective feasibility study. BMJ open. 2018;8(6):e020321.

35. Tomasino KN, Lattie EG, Ho J, Palac HL, Kaiser SM, Mohr DC. Harnessing peer support in an online intervention for older adults with depression. The American Journal of Geriatric Psychiatry. 2017;25(10):1109-19.

36. Shamsikhani S, Ahmadi F, Kazemnejad A, Vaismoradi M. Typology of Family Support in Home Care for Iranian Older People: A Qualitative Study. International Journal of Environmental Research and Public Health. 2021;18(12):6361.

37. Wright KB. Communication in health-related online social support groups/communities: A review of research on predictors of participation, applications of social support theory, and health outcomes. Review of Communication Research. 2016;4:65-87.

38. Pantridge CE, Charles VA, DeHart DD, Iachini AL, Seay KD, Clone S, et al. A qualitative study of the role of peer support specialists in substance use disorder treatment: Examining the types of support provided. Alcoholism Treatment Quarterly. 2016;34(3):337-53.

39. Mercan Y, Selcuk K, Sayılan A. The relationship between types of physical disabilities and the Instrumental Activities of Daily Living (IADL) in the elderly. Family Medicine &amp; Primary Care Review. 2021;23(1):22-8.

40. Creswell JW, Clark VLP. Designing and conducting mixed methods research: Sage publications; 2017.

41. medicine JH. Types of Home Health Care Services 2022 [Available from: <https://www.hopkinsmedicine.org/health/caregiving/types-of-home-health-care-services>.

42. Halter J, Ouslander J, Tinetti M, Studenski S, High K, Asthana S. Hazzard's geriatric medicine and gerontology: McGraw-Hill Prof Med/Tech; 2009.

43. Sunderland K, Mishkin W. Guidelines for the practice and training of peer support. 2013.

44. Musiał K, Kazienko P. Social networks on the internet. World Wide Web. 2013;16(1):31-72.

45. Sodani M, Shogaeyan M, Neysi A. The effect of group logo-therapy on loneliness in retired men. Research in Cognitive and Behavioral Sciences. 2012;2(1):43-54.

46. Russell DW. UCLA Loneliness Scale (Version 3): Reliability, validity, and factor structure. Journal of personality assessment. 1996;66(1):20-40.

47. Hyde M, Wiggins RD, Higgs P, Blane DB. A measure of quality of life in early old age: the theory, development and properties of a needs satisfaction model (CASP-19). Aging & mental health. 2003;7(3):186-94.

48. Heravi-Karimooi M, Rejeh N, Garshasbi A, Montazeri A, Bandari R. Psychometric properties of the Persian version of the quality of life in early old age (CASP-19). Iranian Journal of Psychiatry and Behavioral Sciences. 2018;12(2).

49. Goldberg DP, Hillier VF. A scaled version of the General Health Questionnaire. Psychological medicine. 1979;9(1):139-45.

50. Namjoo S, Shaghaghi A, Sarbaksh P, Allahverdipour H, Pakpour AH. Psychometric properties of the General Health Questionnaire (GHQ-12) to be applied for the Iranian elder population. Aging & mental health. 2017;21(10):1047-51.

51. Zimet GD, Dahlem NW, Zimet SG, Farley GK. The multidimensional scale of perceived social support. Journal of personality assessment. 1988;52(1):30-41.

52. Nakhodaeezadeh M, Jafarabadi MA, Allahverdipour H, Matlabi H, Dehkordi FR. Home environment and its relation with quality of life of older people. Journal of Housing for the Elderly. 2017;31(3):272-85.

53. Süderhamn O, Ek A-C, Pürn I. The self-care ability scale for the elderly. Scandinavian Journal of Occupational Therapy. 1996;3(2):69-78.

54. Tabrizi JS, Behghadami MA, Saadati M, Söderhamn U. Self-care ability of older people living in urban areas of northwestern Iran. Iranian journal of public health. 2018;47(12):1899.

55. Lubben J, Blozik E, Gillmann G, Iliffe S, von Renteln Kruse W, Beck JC, et al. Performance of an abbreviated version of the Lubben Social Network Scale among three European community-dwelling older adult populations. The Gerontologist. 2006;46(4):503-13.

56. Rahemi Z, Dunphy LM, Newman D. Preferences regarding and communication about end-of-life care among older Iranian-American adults. Western journal of nursing research. 2019;41(10):1499-516.

57. Hojati H, Sharif-Nia S-H, Pur H-a, Nik-Khah F, Asayesh H. The effect of reminiscence groups on loneliness and the need for belonging in elders. Journal of Health and Care. 2011;13(1):0-.

58. Gholami J, Shaghaghi S, Nahidiazar F. Urban landscape in informal Settlements of Iran Case study Golestan province (Aliabad-e Katul). Advances in Environmental Biology. 2013;7(11):3271-8.

59. Buus N, Perron A. The quality of quality criteria: Replicating the development of the Consolidated Criteria for Reporting Qualitative Research (COREQ). International journal of nursing studies. 2020;102:103452.

60. Elo S, Kyngäs H. The qualitative content analysis process. Journal of advanced nursing. 2008;62(1):107-15.

61. Tesch R. Qualitative research: Analysis types and software: Routledge; 2013.

62. Lindgren B-M, Lundman B, Graneheim UH. Abstraction and interpretation during the qualitative content analysis process. International journal of nursing studies. 2020;108:103632.
